# hnRNP A1 in RNA metabolism regulation and as a potential therapeutic target

**DOI:** 10.3389/fphar.2022.986409

**Published:** 2022-10-21

**Authors:** Jianguo Feng, Jianlong Zhou, Yunxiao Lin, Wenhua Huang

**Affiliations:** ^1^ Guangdong Engineering Research Center for Translation of Medical 3D Printing Application, Guangdong Provincial Key Laboratory of Medical Biomechanics, School of Basic Medical Sciences, Southern Medical University, Guangzhou, China; ^2^ Affiliated Xinhui Hospital, People’s Hospital of Xinhui District, Southern Medical University, Jiangmen, Guangdong Province, China; ^3^ Laboratory of Anesthesiology, Affiliated Hospital of Southwest Medical University, Luzhou, Sichuan Province, China; ^4^ Department of Oncology, Guangxi International Zhuang Medicine Hospital, Nanning, China

**Keywords:** alternative splicing, hnRNP A1, RNA binding protein, RNA metabolism, splicing factor

## Abstract

Abnormal RNA metabolism, regulated by various RNA binding proteins, can have functional consequences for multiple diseases. Heterogeneous nuclear ribonucleoprotein A1 (hnRNP A1) is an important RNA binding protein, that regulates various RNA metabolic processes, including transcription, alternative splicing of pre-mRNA, translation, miRNA processing and mRNA stability. As a potent splicing factor, hnRNP A1 can regulate multiple splicing events, including itself, collaborating with other cooperative or antagonistical splicing factors by binding to splicing sites and regulatory elements in exons or introns. hnRNP A1 can modulate gene transcription by directly interacting with promoters or indirectly impacting Pol II activities. Moreover, by interacting with the internal ribosome entry site (IRES) or 3′-UTR of mRNAs, hnRNP A1 can affect mRNA translation. hnRNP A1 can alter the stability of mRNAs by binding to specific locations of 3′-UTR, miRNAs biogenesis and Nonsense-mediated mRNA decay (NMD) pathway. In this review, we conclude the selective sites where hnRNP A1 binds to RNA and DNA, and the co-regulatory factors that interact with hnRNP A1. Given the dysregulation of hnRNP A1 in diverse diseases, especially in cancers and neurodegeneration diseases, targeting hnRNP A1 for therapeutic treatment is extremely promising. Therefore, this review also provides the small-molecule drugs, biomedicines and novel strategies targeting hnRNP A1 for therapeutic purposes.

## Introduction

hnRNPs family consists of RNA-binding proteins, that includes at least 20 members named from A to U (1). hnRNP A1 belongs to the hnRNPA/B subfamily and is one of the most abundant and broadly expressed nuclear proteins. HnRNP A1 is first identified as one of the core proteins of ribonucleoprotein complexes (Choi and Dreyfuss, 1984; Lothstein et al., 1985; Wilk et al., 1985; Han et al., 2010). Subsequently, the RNA-binding ability (Schenkel et al., 1988) and the gene alternative splicing regulatory roles (Biamonti et al., 1989) of hnRNP A1 are observed. Intensive studies have revealed the role of hnRNP A1 in regulating normal physiological functions and pathologic processes (Roy et al., 2017; Clarke et al., 2021).

As a splicing factor, hnRNP A1 can modulate the splicing of crucial genes to produce specific protein variants, contributing to human diseases such as tumorigenesis and neurological diseases. Elevated hnRNP A1 levels in cancer cells attenuate cell apoptosis, by regulating the gene splicing process to generate specific protein variants (Kedzierska and Piekielko-Witkowska, 2017). hnRNP A1-mediated alternative splicing of genes in the brain causes severe mental disorders (Donev et al., 2007; Babic et al., 2013; Bruun et al., 2018; Beijer et al., 2021). hnRNP A1 also shows multiple physiological functions on cell proliferation (Yang et al., 2019), cell survival (Feng et al., 2018), cell cycle (Yu et al., 2015), cell migration, cell stemness, cellular senescence (Shimada et al., 2009), etc.

In addition to regulating mRNA alternative splicing events, hnRNP A1 involved in gene transcription, internal ribosomal entry sites (IRES)-dependent mRNA translation, mRNA transportation, mRNA stability and microRNA biogenesis. This review article describes our current understanding of hnRNP A1’s underlying mechanisms regulating RNA metabolism and provides existing approaches targeting hnRNP A1 or its functions.

## The structure of hnRNP A1

**FIGURE 1:**
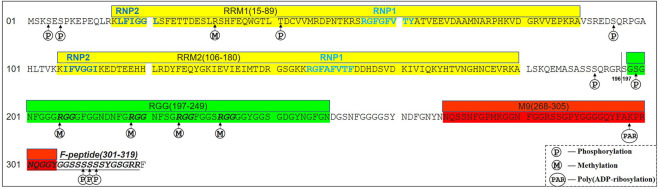
A scheme of the hnRNP A1 primary amino acids sequence (UniProt:P09651-2). The amino acids (1–196) are the N-terminus (also named UP-1) of hnRNP A1, consisting of two RNA recognition motifs (RRM1 and RRM2, Yellow). The amino acids sequences of dark gray are the C-terminus (also named GRD,197–320), composed of RGG (197–249, Green) with four RGG motifs (Italic Bold), M9 (268–305, Red) and F-peptide (301–319, Italic Underline). The phosphorylation and poly (ADP-ribosylation) sites of hnRNP A1 are marked.

The hnRNP A1 gene is located at 12q13.13, consisting of 10 exons and 9 introns. The alternative splicing of pre-mRNA hnRNP A1 generates an alternate in-frame exon (exon 7b, 52 amino acids), resulting in an extended protein hnRNP A1B (372 amino acids, UniProt:P09651) compared to hnRNP A1 (320 amino acids, UniProt:P09651-2) (Hutchison et al., 2002). The hnRNP A1 isoform is much more abundant than hnRNP A1B isoform. The N-terminus domain (also named UP-1, 1–196 amino acids) of hnRNP A1 comprises two RNA recognition motifs (RRMs) with highly similar sequences, PRM1 and PRM2, which consist of four β-sheets, two α-helices (βαββαβ), conserved RNP1 octameric and RNP2 hexameric about 30 amino acid residues apart (Dreyfuss et al., 1988; Xu et al., 1997; Ding et al., 1999). The C-terminal of hnRNP A1 is a glycine-rich region (also called Glycine-rich domain, 197–320 amino acids), with an RGG box (197–249 amino acids) within four RGG repeats followed by nuclear localization signal (NLS) M9 domain (268–305 amino acids) and F peptide (301–319 amino acids) with six consecutive serines (S308-S313) (Figure 1) (Izaurralde et al., 1997; Allemand et al., 2005; Ghosh and Singh, 2018).

The two RRMs of hnRNP A1 are the common RNA-binding domains recognizing and binding to splicing regulatory elements to regulate gene alternative splicing events (Beusch et al., 2017). In addition, the two RBMs also contribute to RNA packaging and trafficking (Shamoo et al., 1995; Shan et al., 2000), and bind to single-stranded telomeric DNA (21). The Glycine-rich domain of hnRNP A1 mediates protein-protein interactions and RNA binding (Bekenstein and Soreq, 2013). The RGG box in Glycine-rich domain, which consists of four RGG repeats, has been shown to mediate self-interaction, and interaction with other hnRNPs or RNA binding proteins including serine-arginine (SR) proteins (Cartegni et al., 1996; Fisette et al., 2010). It can also recognize the telomere G-quadruplex DNA (22), as well as affect its internal ribosome entry site trans-acting factor (ITAF) activity (Wall and Lewis, 2017). M9 is a 38 amino acid-long motif, downstream of the RGG box, which is closely related to the cellular localization of hnRNP A1 and plays a key role in mRNA nuclear export and protein nuclear import (Siomi and Dreyfuss, 1995; Izaurralde et al., 1997), whereas hnRNP A1’s regulatory functions on the mRNA stability and translational activity are depended on its subcellular location. Furthermore, F-peptide is located at the C-terminal domain, adjacent to the M9 motif, and its phosphorylation status and can influence the rate of hnRNP A1 nuclear import (Allemand et al., 2005).

## The function of hnRNP A1 in alternative splicing

Alternative splicing is a pre-mRNA process regulated by *cis*-acting elements and *trans*-acting factors. The *cis*-acting elements contain exonic splicing enhancer/silencer (ESE/ESS) and intronic splicing enhancer/silencer (ISE/ISS). These splicing enhancers or splicing silencers interact with *trans*-acting factors, conferring a positive or negative effect on splice site recognition by the spliceosome, ultimately influencing splicing outcome (Zhu et al., 2001). While the *trans*-acting factors comprise two major classes of splicing factors, hnRNPs and SR proteins (Busch and Hertel, 2012). hnRNP A1, one of the most important hnRNPs splicing factors, regulates alternative splicing in numerous mammalian genes including the caspase-2 gene, c-src, SMN2 gene, and even itself (Suzuki and Matsuoka, 2017). Moreover, the hnRNP A1 participates in the alternative splicing process of several genes in virus harboring Human Immunodeficiency Virus-1 (HIV-1) (Damgaard et al., 2002; Marchand et al., 2002; Zahler et al., 2004), Human papillomavirus (HPV) (Ajiro et al., 2016) and Human T-lymphotropic virus type 1(HTLV-1) (Princler et al., 2003), indicating that both viral RNA *cis*-elements and host splicing factors govern virus pre-mRNA alternative splicing (Kaur and Lal, 2020).

hnRNP A1 is associated with spliceosome assembly, then in the two consecutive transesterification reactions that lead to excision of the introns and joining of the exons (Jurica et al., 2002; Zhou et al., 2002). Finally, by establishing a complex with U2AF, hnRNP A1 causes the spliceosome to select functional 3′ splicing sites (Tavanez et al., 2012). hnRNP A1 can bind to exon splicing silencers (Del Gatto-Konczak et al., 1999), intron binding sites (Expert-Bezancon et al., 2004) or splice sites (Chiou et al., 2013) to repress exon splicing, suggesting that hnRNP A1 may act as a splicing repressor (Fisette et al., 2010). However, it can also have beneficial effects on exon splicing of several genes, including CDK2 (16), Fas (Oh et al., 2013) and IRF3(49). The alternative splicing regulatory functions of hnRNP A1, including how it interacts with the specific binding sites or other co-regulatory splicing factors, will be discussed in detail as following.

### RNA specific binding sites of hnRNP A1

hnRNP A1 is an RNA-binding protein that binds to particular sequence, that Burd et al. (Burd and Dreyfuss, 1994) identified the high affinity hnRNP A1 binding sites, UAGGGA/U, using the systematic evolution of ligands by exponential enrichment (SELEX) experiment. Crosslinking immunoprecipitation followed by high throughput sequencing (CLIP-seq) analyses showed preferential binding of hnRNP A1 to UAGU sequence element (Huelga et al., 2012). Furthermore, the individual-nucleotide resolution crosslinking immunoprecipitation (iCLIP) identified UAGG as the hnRNP A1 binding motif (Bruun et al., 2016). The positions where hnRNP A1 interacts with its binding sequence may affect the consequences of the alternative splicing events. According to the study from Burd et al. (Burd and Dreyfuss, 1994), the ‘winner’ sequence containing a duplication of this UAGGGA/U sequence separated by two nucleotides showed the highest affinity for hnRNP A1, where the binding consensus resembled the 5′ and 3′ splice sites. The UAGGGC sequence at the E9 5′ splice site of the *PKM* gene has been identified as hnRNP A1’s binding site, facilitating the alternative splicing process that results in PKM2 isoform (David et al., 2010). In the alternative splicing process of the *MAPT* gene, hnRNP A1 binds to the sequence caaagGTGC at the 3′ splice site of exon 10 to promote exon 10 skipping (Liu et al., 2020). Another strong hnRNP A1 binding sequence (GAGGAAG) at 5′ splice site of exon 5 interacted with hnRNP A1, enabling Fas gene inclusion in distal exon 6 (Oh et al., 2013). These findings suggest that hnRNP A1 regulates the splicing by binding to a particular sequence in splice sites.

In addition to the splice sites, hnRNP A1 can bind splicing regulatory elements, such as splicing silencers in exons or introns, to regulate the alternative splicing of various genes. For exon splicing regulation, ESSs suppress exon inclusion by blocking the exon splice sites, while ESEs increase exon inclusion by recruiting SR splicing factors (Graveley, 2000). ISSs hinder exon inclusion by recruiting repressors, while ISEs antagonistically promote exon inclusion (Wang and Burge, 2008). However, the effects of a single regulatory element on alternative splicing events can have two opposing consequences depending on its locations and binding factors (Wang et al., 2012). hnRNP A1 regulates gene splicing by binding to splicing silencers in exons and introns. The binding of hnRNP A1 to the ESS sequence (TGCGGC) in *Ron* exon 12 is relevant for its ability to promote Ron exon 11 inclusion, contributing to mesenchymal-to-epithelial transition of cancer cells (Bonomi et al., 2013). Furthermore, the hnRNP A1 inhibits exon 7 inclusion during gene splicing by binding both ESSs at exon 7 and ISS N1 at intron 7 of SMN2, that suppressing the regulatory effects of hnRNP A1 on SMN2 alternative splicing leads to a functional protein with exon 7 inclusion beneficial in treating spinal muscular atrophy (Kashima et al., 2007a; Beusch et al., 2017). Besides promoting exon exclusion, hnRNP A1 can also promote exon inclusion by splicing. With binding to ESS sites in exon 12, hnRNP A1 increases exon 12 inclusion of ATP7B, that hnRNP A1 silencing promotes ATP7B exon12 exclusion, potentially attenuating the toxic effects of ATP7B exon 12 mutation in Wilson’s disease (Lin et al., 2015). Similarly, overexpression of hnRNP A1 causes CDK2 to be included in exon 5, promoting cell cycle progression of oral squamous cell carcinoma (Yu et al., 2015), Fas to be included in exon 6 protecting cells from apoptosis (Oh et al., 2013), and so on. The binding sites and modulated splicing events of hnRNP A1 are listed in Table 1.

**TABLE 1:** The alternative splicing events regulated by hnRNP A1.

Genes	Binding sites	Isoforms	Co-regulatory factors	Diseases/Tissues/Cells/Virus	Reference
APOL1	a consensus *cis*-acting element in exon 4	Promoting exon 4 exclusion	—	Human glomerular and tubular cells	Cheatham et al. (2018)
APP	Alu element in introns 6 and 8	Promoting exons 7 and 8 skipping	SRSF2(Cooperative)	NT2N neuronal cells	Donev et al. (2007)
AR	UAGGGA in splice sites	Promoting AR-V7 expression	—	Prostate cancer	Nadiminty et al. (2015)
ATP7B	ESS sites in exon12	Promoting exon 12 inclusion	—	Wilson’s diseases	Lin et al. (2015)
ATM	Alu-derived Intronic Splicing enhancer (ISE) in intron 20	Promoting cryptic exon exclusion	DAZAP1 (Antagonistic)	HeLa cell	Pastor and Pagani, (2011)
Bcl-x	5′ splice site	Promoting Bcl-x(S) expression	(hnRNP) F/H/Sam68 (Cooperative)	HEK293 cell	Cloutier et al. (2018)
beta-tropomyosin	G-rich intronic sequence (S3) downstream of exon 6B	Promoting exon 6B exclusion	SRSF1, SRSF2 (Antagonistic); hnRNP F/H (Cooperative)	HeLa cell	Expert-Bezancon et al. (2004)
CCDC50	—	Promoting exon 6 skipping	—	Clear cell renal cell carcinoma	Sun et al. (2020)
CD44	Splice regulatory elements in exon v5	Promoting exon v5 exclusion	—	CB3 and NIH-3T3 cells	Matter et al. (2000)
CD44	—	Increasing c5v6v7v8v9v10c6 and c5v6v8v9v10c6; inhibiting c5v6c6	—	MCF7, MCF10A and MDA-MB-231 cells	Loh et al. (2015)
CDK2	GUAGUAGU in intron 4	Promoting exon 5 inclusion	—	Oral squamous cell carcinoma	Yu et al. (2015)
CEACAM1	3′ to exon 7	Promoting exon 7 exclusion	hnRNP L (Cooperative)	ZR75 and MDA-MB-468 cells	Dery et al. (2011)
			hnRNP M (Antagonistic)		
CFTR	ISS of intron 9	Promoting exon 9 skipping	SRSF1, SRSF5, SRSF6, SRSF4 (Cooperative)	Hep3B cell	Pagani et al. (2000)
c-H-ras	intronic silencer sequence (rasISS1) of intron D2	Promoting intron D1 exclusion	SRSF2, SRSF5 (Antagonistic)	HeLa cell	Guil et al. (2003)
c-src	3′ splice site of exon N1	Promoting exon N1 exclusion	SRSF1, SRSF2 (Antagonistic) hnRNP I (Cooperative)	HeLa and WERI-1 cells	Rooke et al. (2003)
Fas	GAGGAA at 5′ splice site of exon 5	Promoting exon 6 inclusion	—	MDA-MB-231, HeLa and HCT116 cells	Oh et al. (2013)
FGFR2	K-SAM ESS	Promoting K-SAM exon skipping	TIA-1 (Antagonistic)	HEK293 cell	Gesnel et al. (2009)
Gαs	—	Promoting exon 3 skipping	SRSF1(Antagonistic)	Myometrial smooth muscle cells	Pollard et al. (2002)
GLA	ESS overlapping the 5′ splice site	Preventing pseudoexon inclusion	hnRNP A2/B1 (Cooperative)	HeLa and HepG2 cells	Palhais et al. (2016)
GRIN1 gene	exonic UAGGs and the 5′-splice-site proximal GGGG motif	Promoting CI cassette exon (exon 19) skipping	hnRNP H (Antagonistic)	Rat cortical culture; PC12 and C2C12 cells	(Han et al., 2005; An and Grabowski, 2007)
HER2	—	Promoting intron 8 retention	—	SKBR3 cell	Silipo et al. (2017)
HIV-1 rev/tat	intronic splicing silencer (ISS), a novel UAG motif in the exon splicing enhancer (ESE), and the exon splicing silencer (ESS3)	Inhibiting the second intron exclusion	SRSF1, SRSF2(Antagonistic)	HIV-1	(Damgaard et al., 2002; Marchand et al., 2002; Zahler et al., 2004)
HN1	—	Promoting alternative polyadenylation (APA) -3′ UTR shortening	—	HEK293, HUVEC and A549 cells	Jia et al. (2019)
HMGCR	—	Promoting exon 13 skipping	—	HepG2, Hep3B and Huh7 cells	Yu et al. (2014)
HPV18	ESS at the E7	Preventing HPV18 233^416 splicing in the E6 ORF	—	HPV	Ajiro et al. (2016)
HipK3	HipK3-T purine-rich region	Promoting HipK3-T exclusion	Tra2β-1 (Antagonistic)	Testis	Venables et al. (2005)
hnRNP A1	—	Promoting intron 10 inclusion	Autoregulation	HeLa and NSC34 cells	Suzuki and Matsuoka, (2017)
hnRNP A1	the CE1a and CE4 elements	Promoting exon 7B skipping	hnRNP A2 (Cooperative)	HeLa, CB3C7, and CB3C7-20 cells	Hutchison et al. (2002)
IKBKAP	ISS of intron 20; ESS1 and ESS2 of exon 20	Promoting exon 20 skipping	—	Familial dysautonomia	Bruun et al. (2018)
IRF-3	(UAGGGA) binding motifs in intron 1	Promoting exons 2 and 3 inclusion	SRSF1 (Cooperative)	A549 and Calu-6 cells	Guo et al. (2013)
Ich-1	—	Promoting exon 9 (61bp) inclusion	SRSF1, SRSF2(Antagonistic)	HeLa cell	Jiang et al. (1998)
INSR	AGGGA sites in intron 10	Promoting exon 11 skipping	hnRNP F (Antagonistic)	HeLa, HepG2 and HEK293 cells	Talukdar et al. (2011)
LOXL4	—	Promoting exon 9 skipping	—	ES-2 and MDA-MB-231 cells	Sebban et al. (2013)
Mag	UAGGU at the 5′ splice site of Mag exon 12	Promoting (S-MAG) exon 12 skipping	—	Mouse brainstem; HeLa and CG4 cells	(Zhao et al., 2010; Zearfoss et al., 2013)
MLCK	UAGGGA in Intron 10	Promoting exon 11 skipping	—	Human pulmonary artery endothelial cells	Mascarenhas et al. (2018)
Max	intronic region in intron 4	Promoting exon 5 inclusion	—	Glioblastoma	Babic et al. (2013)
Mdm2	—	Promoting exon3-10 skipping	—	HaCaT cell	Feng et al. (2016)
PKM	Intronic UAGGGC sequence flanking exon 9	Repressing the use of exon 9 to generate PKM2 (exons 9 and 10 mutually splicing)	hnRNP A2, hnRNP I (Cooperative)	brain and glioma samples; NIH-3T3, C2C12 cells	David et al. (2010)
PKM	—	Repressing the use of exon 9 to generate PKM2 (exons 9 and 10 mutually splicing)	hnRNP A2, hnRNP I (Cooperative)	HeLa, HEK293, U-118MG, A-172, SK-N-BE and C2C12 cells	Clower et al. (2010)
PKM	Intronic UAGGGC sequence flanking exon 9	Repressing the use of exon 9 to generate PKM2 (exons 9 and 10 mutually splicing)	SAM68(Cooperative)	Lung adenocarcinoma	Zhu et al. (2021)
PKM	Exon9-Intron9; Exon9; Exon10	Repressing the use of exon 9 to generate PKM2 (exons 9 and 10 mutually splicing)	SRSF3(Cooperative)	Colon cancer DLD-1 and WiDr Cells	Kuranaga et al. (2018)
PKM	—	Repressing the use of exon 9 to generate PKM2 (exons 9 and 10 mutually splicing)	NEK2(Cooperative)	Multiple Myeloma Cells	Gu et al. (2017)
PKM	—	Repressing the use of exon 9 to generate PKM2 (exons 9 and 10 mutually splicing)	HIF1 (Cooperative)	Mouse cardiac muscle	Williams et al. (2018)
PKM	—	Repressing the use of exon 9 to generate PKM2 (exons 9 and 10 mutually splicing)	RBM4 (Antagonistic)	Neuronal differentiation of MSCs	Su et al. (2017)
PKM	Intronic UAGGGC sequence flanking exon 9	Repressing the use of exon 9 to generate PKM2 (exons 9 and 10 mutually splicing)	RBMX (Antagonistic)	Bladder cancer	Yan et al. (2021)
p53-inducible gene 3 (PIG3)	ESS in exon 4	Promoting exon 4 skipping	—	HeLa and MCF7 cells	Nicholls and Beattie, (2008)
RAGE	—	Inhibiting alternative inclusion of part of intron 9 and removal of exon 10 to form flRAGE	Tra2β-1 (Antagonistic)	SH-SY5Y cell	Liu et al. (2015)
Rac1	UAAAGA within exon 3b	Promoting exon 3b exclusion	—	SCp2 and EpH4 cells	Pelisch et al. (2012)
Ron	Splicing silencer of exon 12	Inhibiting Ron exon 11 skipping (Δ Ron)	SRSF1(Antagonistic)	HeLa, KATOⅢ and MDA-MB-435S cells	Bonomi et al. (2013)
SMN2	intronic splicing silencer ISS-N1 at the beginning of intron 7	Promoting exon 7 skipping	Tra2β-1 (Antagonistic) hnRNP I (Cooperative)	Spinal muscular atrophy	(Kashima and Manley, 2003; Kashima et al., 2007b; Beusch et al., 2017)
Smad2	—	Promoting exon 9 skipping	Rpl22(Cooperative)	Zebrafish embryos	Zhang et al. (2017)
tau	3′ splice site of exon 10	Promoting exon 10 skipping; intron 9 exclusion	—	SH-SY5Y and HEK293T cells	Liu et al. (2020)
Tid1	—	Promoting exon 11 skipping	hnRNP A2 (Cooperative)	Non-small cell lung cancer	Chen et al. (2016)
TIMP1	3′ splice site of exon 4	Promoting intron 3 retention	—	HCT116 cell	Flodrops et al. (2020)
TRA2B	—	Promoting exon 2 inclusion	hnRNP U (Antagonistic)	HCT116 and HCEC-1CT cells	Nishikawa et al. (2019)

hnRNP A1 binding to both the splicing sites and splicing regulatory elements is required for the splicing of some certain genes, such as *Insulin Receptor (INSR)* gene. In the alternative splicing of *INSR* gene, hnRNP A1 binds to both the 5’ splice site of intron 11 and the ISE site of intron 10, preventing the inclusion of exon 11 in INSR gene splicing, finally influencing the glucose metabolism (Talukdar et al., 2011). Besides, the alternative splicing regulatory activities of hnRNP A1 were dependent not only on the RNA-binding properties but also on protein-protein interactions with other splicing factors (Cartegni et al., 1996). As mentioned above, the selective binding is mostly mediated by the Gly-rich C-terminal region. The hnRNP A1-interacted proteins responsible for alternative splicing regulation can be sorted into two categories: cooperative and antagonistic splicing factors.

### Alternative splicing regulation by hnRNP A1 with cooperative splicing factors

hnRNP A1 participates in spliceosome assembly, and can interact with other splicing factors and RBPs to regulate alternative splicing of genes cooperatively (Jurica et al., 2002; Zhou et al., 2002). Firstly, the homotypic interactions of hnRNP A1 *via* its UP1 domain have been documented using the bioluminescence resonance energy transfer (BRET) technology (Ding et al., 1999; Fisette et al., 2010). Like hnRNP A1, the longer isoform hnRNP A1B also exhibits homotypic interactions (Gueroussov et al., 2017; Gagne et al., 2021). Other RBPs, such as hnRNP C and TDP-43, have been shown to dimerize, suggesting that dimerization could affect their functions (Shiina et al., 2010; Cienikova et al., 2015). Secondly, hnRNP A1 cooperates with other hnRNPs family members to modulate gene alternative splicing. hnRNP A1B-hnRNP A1 heterodimer was observed in neurons (Gagne et al., 2021). The heterotypic interactions between hnRNP H and hnRNP A1 in live cells can be captured in live cells through BRET signals mediated by the C-terminal of hnRNP A1 and H (Fisette et al., 2010). hnRNP A1 and hnRNP H can collaborate in modulating 5′ splice site selection to further regulate alternative splicing of genes containing Bcl-x (Cloutier et al., 2018), beta-tropomyosin (Expert-Bezancon et al., 2004), and viral genes (Stoltzfus and Madsen, 2006). By regulating the alternative splicing of PKM gene, hnRNP A1, hnRNP A2 and hnRNPI (also known as PTB) worked together to enhance PKM2 expression (David et al., 2010). SAM68’s 351–443 aa region binds to the RGG motif of hnRNP A1, causing hnRNP A1-dependent PKM splicing to promote oncogenic PKM2 isoform formation while inhibiting PKM1 isoform formation. For c-src exon N1 splicing regulation, hnRNP A1 collaborates with hnRNP I to play key regulatory roles for exon exclusion by binding to 3’splice site of exon N1. The removal of hnRNP I binding sites in N1 Exon inhibits its effects of splicing repression but does not influence hnRNP A1-modulated N1 exon skipping. Thus, the impact of hnRNP A1 and hnRNP I on c-src splicing regulation are cumulative rather than synergistic (Rooke et al., 2003). hnRNPs such as hnRNP F (66), hnRNP A2/B1 (69) and others, as shown in Table 1 have been determined to collaborate with hnRNP A1 modulating gene alternative splicing. Thirdly, several SRs could cooperate with hnRNP A1 in regulating splicing events, although SR proteins often compete with hnRNPs (Table 1). These findings suggest that the regulation of alternative splicing events by hnRNP A1 still requires the involvement of multiple splicing factors including hnRNPs and SRs.

### Alternative splicing regulation by hnRNP A1 with antagonistic splicing factors

Numerous splicing factors have been identified as hnRNP A1 antagonists in regulating alternative splicing. SR proteins are non-snRNP prtoteins involved in both constitutive and the controlled splicing processes (Graveley et al., 2001). SR splicing factors are the most predominant splicing factors antagonistically interacting with hnRNP A1 to modulate the splicing process. SRSF1 (ASF/SF2) is a well-established hnRNP A1 antagonist (Mayeda et al., 1993). According to the study from Mayeda et al. (Mayeda and Krainer, 1992), when alternative 5′ splice sites are present in pre-mRNAs, the relative concentration of hnRNP A1 and the crucial splicing factor SRSF1 influence the selection of 5′ splice site. Instead of having substrate-specific effects, these splicing factors have a shared effect on the polarity of alternative 5′ splice-site selection. When the concentration of SRSF1 is higher than hnRNP A1, it will lead to activation of proximal 5′ splice sites. While when the hnRNP A1 expression level is higher than SRSF1, it will benefit the activation of distal 5’ splice sites (Mayeda and Krainer, 1992). In exon 3 HIV-1 tat pre-mRNA, researchers investigated that hnRNP A1 and SR proteins participate in the antagonistic effects between ESE and ESS(33). hnRNP A1 inhibits tat23 splicing by cooperating along the exon, beginning at ESS3. SRSF1 reverses the process and leads to exon recognition, while SRSF2 (SC35) is ineffective (Zhu et al., 2001). The SRSF2-dependent splicing could be specifically blocked and mediated by the hnRNP A1 binding motif UAGUGAA in ESS3(33). Several other SRs and SR-like splicing factors that serve as hnRNP A1 antagonists have also been discovered (Jiang et al., 1998; Pollard et al., 2002; Guil et al., 2003; Rooke et al., 2003; Expert-Bezancon et al., 2004; Venables et al., 2005; Bonomi et al., 2013; Liu et al., 2015).

Furthermore, under certain circumstances, several hnRNPs splicing factors may have the opposite effect on hnRNP A1 on alternative splicing regulation. By binding to a particular motif positioned centrally and 3′ to exon 7, hnRNP A1 promotes exon 7 exclusion of CEACAM1 gene, while overexpression of hnRNP M causes exon 7 inclusion (Dery et al., 2011). Similarly, hnRNP H (79, 80) and hnRNP F (61) antagonize the activity of hnRNP A1 on the exon skipping in the splicing events of *GRIN1* and *INSR* genes. Interestingly, hnRNP A1 promotes exon 2 inclusion of Tra2β, while hnRNP U facilitates its skipping, and further ectopic overexpression or deletion experiments reveal that hnRNP A1 and hnRNP U also influence the transcription of TRA2B, suggesting important roles of hnRNP A1 and hnRNP U in the coupling between gene transcription and alternative splicing, as they have both DNA- and RNA-binding abilities (Nishikawa et al., 2019).

There is antagonism between various types of splicing factors and hnRNP A1 in addition to the hnRNPs and SRs proteins. RBMX can competitively bind to the RGG motif of hnRNP A1, blocking the binding of hnRNP A1 to the sequences flanking PKM exon 9, suppressing the formation of PKM2 (82). RNA binding protein TIA-1 promotes K-SAM exon inclusion of *FGFR2* through binding to the downstream intron, while hnRNP A1 represses the exon splicing by binding to the exon (Gesnel et al., 2009).

**FIGURE 2:**
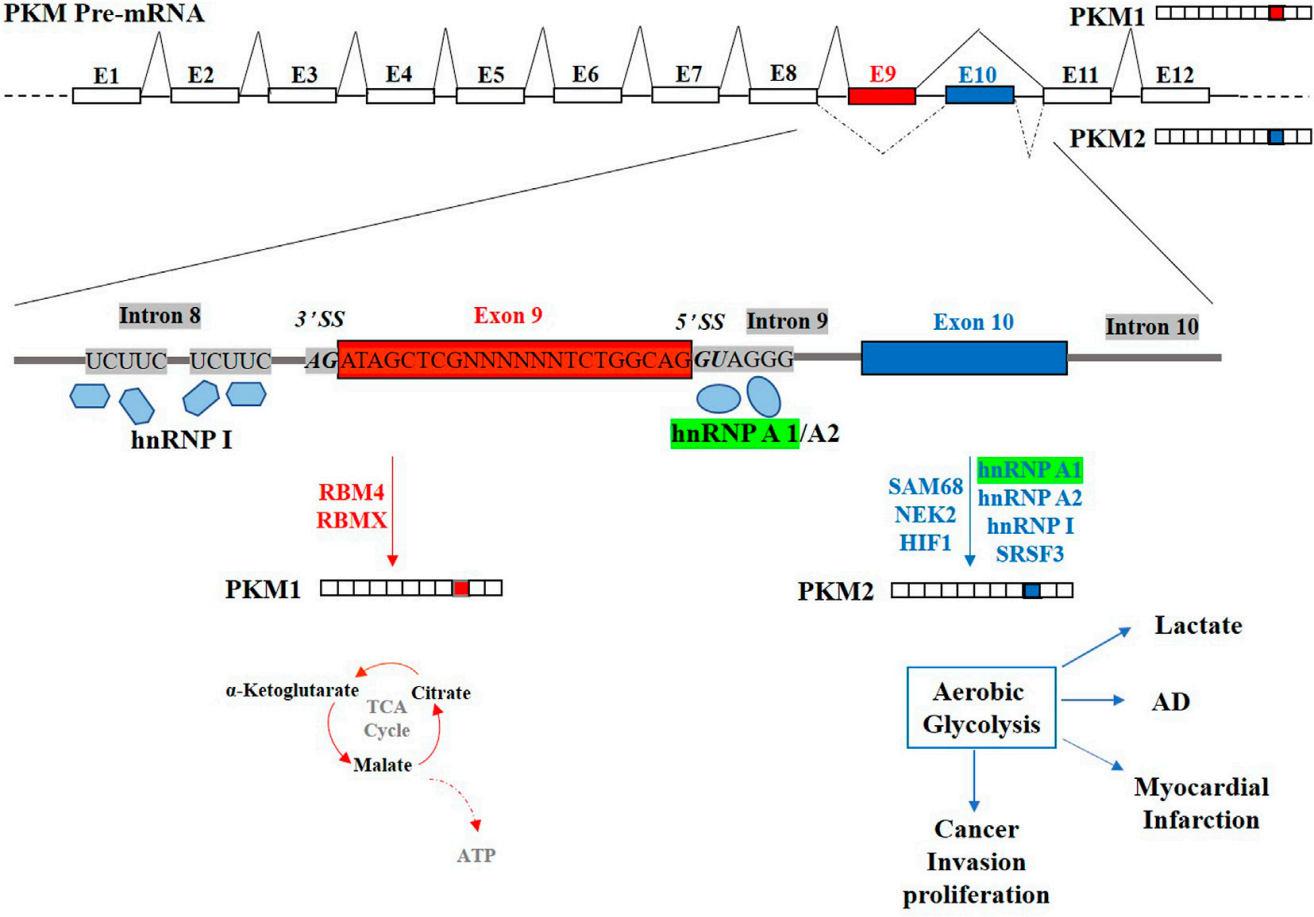
Schematic representation of the human pre-PKM gene and the regulation of its mutually exclusive alternative splicing. Exon9 (E9) and Exon10 (E10) of PKM gene are two mutually exclusive exons. Generation of the PKM1 isoform using E9 is crucial for tricarboxylic acid cycle (TCA cycle) and oxidative phosphorylation producing maximum ATP. PKM2, including E10 but not E9, results in aerobic glycolysis (the Warburg effect) with high lactate production, contributing to AD, myocardial infarction and cancer progression. hnRNP A1 (Highlighted in Green) suppresses the E9 inclusion by binding to 5′ SS downstream E9 and co-regulation with cooperative factors (hnRNP A2, hnRNP I, SRSF3, SAM68, NEK2 and HIF1) and antagonistic factor (RBMX and RBM4).

Altogether, hnRNP A1 is an essential regulator in alternative splicing. HnRNP A1 can collaborate with other factors to cooperatively and antagonistically regulate alternative splicing by interacting with specific sites, splicing regulatory elements in exons or introns, and specific protein domains. Consider the hnRNP A1-regulated alternative splicing of PKM gene, which is a canonical mutually exclusive alternative splicing (Figure 2). Alternative splicing of the PKM gene to skip exon 9 and include exon 10 generates PKM2 in cancer cells through hnRNP A1/A2 binding to UAGGG at 5’ SS in intron 9 and hnRNP I interacting with two UCUUC in intron 8 (Clower et al., 2010). Besides, RBM4 (85) and HIF1 (Williams et al., 2018) exert regulatory role in alternative splicing of PKM in the brain and heart. Other factors such as SRSF3(87), SAM68(88), NEK2 (89) and RBMX (82) are also involved in the alternative splicing process of the PKM gene *via* interacting with hnRNP A1. The isoform PKM2, results in aerobic glycolysis (the Warburg effect) with high lactate production, contributing to AD, myocardial infarction and cancer progression. The use of exon 9 to generate the PKM1 isoform is crucial for the tricarboxylic acid cycle (TCA cycle) and oxidative phosphorylation producing maximum ATP. hnRNP A1 cooperates with cooperative factors (hnRNP A2, hnRNP I, SRSF3, SAM68, NEK2 and HIF1) and antagonistic factors (RBMX and RBM4) to regulate alternative splicing of PKM gene. The hnRNP A1 can regulate the alternative splicing process of multiple target genes and correspondingly, the alternative splicing process of one single gene is regulated by multiple splicing factors.

In addition to canonical linear splicing of pre-mRNA, the back-splicing has been recently well studied to produce circular RNAs (circRNAs) by the joint of a downstream 5′ splice site and an upstream 3′ splice site. Both hnRNP and SR proteins could regulate circRNA biogenesis (Kramer et al., 2015). Particularly, the characteristics of those RNA binding proteins to bind the introns flanking circRNA and form a dimmer, effectively contribute to the biogenesis of circRNAs (Conn et al., 2015). Accordingly, the engineering circRNA regulators (ECRRs) has been proposed recently by Qi et al. (Qi et al., 2021), to specifically promote circular RNA production by combining sequence-specific RNA binding motifs of human Pumilio 1 (PUF domain) with functional domains that could form dimerization, including the UP1 (RRM) domain of HNRNP A1. The PUF-hnRNP A1 ECRR could significantly promote the circRNA production of the exogenous circRNA minigene reporter circGFP, indicating an important cricRNA biogenesis regulatory role of hnRNP A1. Given the importance of circRNAs in various physiological and pathologic conditions, the effects of hnRNP A1 on back-splicing control and specific circRNA generation are worthy of further exploration.

## hnRNP A1 regulates mRNA transcription and translation

Although hnRNP A1 is a powerful RNA binding protein, its role in transcription regulation has been suggested as it could prevent the transcription elongation factor phospho-TEFb from interacting with its repressor molecule, the 7SK RNA (Barrandon et al., 2007; Van Herreweghe et al., 2007). The hnRNP A1 UP1 domain bound specifically to the SL3 domain of 7SK snRNA, forming the 7SK-hnRNP A1 complex, further influenced the 7SK snRNA bioactivity and P-TEFb availability, affecting the initial transcription and elongation processes (Luo et al., 2021). Downregulation of hnRNP A1 causes the promoter-proximal pausing of RNA polymerase II (Pol II) on TEFb-dependent genes, as well as transcriptional repression, suggesting that hnRNP A1 has a role in controlling transcription elongation by Pol II (Lemieux et al., 2015). Along with the eukaryotic gene, hnRNP A1 can bind to the complementary (-)-strand of the leader RNA and intergenic sequence of mouse hepatitis virus (MHV) RNA, enhancing the viral RNA transcription (Li et al., 1997). Besides, N6-methyladenosine (m6A) modification of RNA, an addition of a methyl group at position N6 of adenosine, can influence RNA transcription, alternative splicing, degradation, and translation. The m6A modification is catalyzed by methyltransferase (“writers”), removed by demethylase (“erasers”) and recognized by m6A binding protein (“readers”). As one of the “readers”, HnRNPs including hnRNPA2/B1 (Alarcon et al., 2015) and hnRNP C (Huang et al., 2021) can recognize the methylation sequence on RNAs. Recently, Kumar et al. (Kumar et al., 2021) discovered that hnRNP A1 could be recruited to the m6A-modified SARS-CoV-2 RNA, and act as a m6A “reader” to promote the transcription.

Also, hnRNP A1 has been shown to possess DNA binding activity, by interacting with G-quadruplex structure in the promoter of genes to promote the gene transcription (Cogoi et al., 2017). hnRNP A1 firmly binds the quadruplex-forming GC-elements upstream of the primary transcription start sites and their higher i-motif conformations, increasing HRAS gene transcription (Miglietta et al., 2015). Table 2 lists the genes transcriptionally regulated by hnRNP A1. Interestingly, hnRNP A1 exerts regulating alternative splicing of some certain genes, such as TRA2B, concomitantly influencing its transcription activity, indicating that hnRNP A1 probably involve in the co-transcriptional splicing modulation (Nishikawa et al., 2019).

**TABLE 2:** The transcription and translation of genes enhanced by hnRNP A1.

Genes	Binding sites	Functions	Reference
Acta2	Promoter	Enhancing Transcription	Huang et al. (2013)
APOE	-219T site in the promoter	Enhancing Transcription	Campillos et al. (2003)
ANXA7	Promoter	Enhancing Transcription	Torosyan et al. (2010)
mouse hepatitis virus (MHV) RNA	MHV(-)-strand leader and IG sequences	Enhancing Transcription	Li et al. (1997)
Tagln	Promoter	Enhancing Transcription	Huang et al. (2013)
TRA2B	G-quadruplex	Enhancing Transcription	Nishikawa et al. (2019)
c-myc	IRES	Enhancing Translation	(Jo et al., 2008; Shi et al., 2016)
cyclin D1	IRES	Enhancing Translation	Jo et al. (2008)
egr2	IRES	Enhancing Translation	Rubsamen et al. (2012)
EV71	IRES	Enhancing Translation	Leong et al. (2015)
FGF-2	IRES	Enhancing Translation	Bonnal et al. (2005)
HIF1a	IRES	Enhancing Translation	(Gao et al., 2017; Zeng et al., 2021)
HRAS	GC-elements	Enhancing Translation	Miglietta et al. (2015)
KRAS	G-quadruplex	Enhancing Translation	Paramasivam et al. (2009)
MELOE-1	IRES	Enhancing Translation	Charpentier et al. (2021)
Nfil3	IRES	Enhancing Translation	Kim et al. (2017)
RON	G-quadruplex in 5′-UTR	Enhancing Translation	Cammas et al. (2016)
sST2	IRES	Enhancing Translation	Kunze et al. (2016)
VRK1	3′-UTR	Enhancing the translation	Ryu et al. (2021)

hnRNP A1 is primarily distributed in the nucleus as RNA binding protein, however, it may translocate to the cytoplasm with specific phosphorylation when expose to growth factors (Kunze et al., 2016) and stress stimuli such as osmotic shock or UV irradiation (van der Houven van Oordt et al., 2000; Feng et al., 2018). Translocation is crucial for translational regulatory functions of hnRNP A1 as it can bind to the internal ribosome entry site (IRES) of mRNA. hnRNP A1 acts as an ITAF, affecting the mRNA translation process, regulating the sterol-regulatory-element-binding protein 1a (SREBP-1a) expression in hepatocytes and hepatoma cells (Damiano et al., 2013), and hence modulating the expression of several enzymes involved in lipid synthesis. In addition to the eukaryotic genes, hnRNP A1 enhances the translation of EV71 and HRV-2 by binding to the IRES (128, 129). In hnRNP A1 driven IRES-dependent EV71 RNA translation process, UP1 domain of hnRNP A1 interacts specifically with the stem-loop II (SLII) of the IRES, required for the next translation (Levengood et al., 2013).

In *Drosophila*, hnRNP A1 binds to the 3′ untranslated region (UTR) of Nanos mRNA, inhibiting Nanos translation, whereas poly (ADP-ribosylation) of hnRNP A1 relieves the translation repression (Ji and Tulin, 2016). hnRNP A1 can bind to the IRES of cyclin D1 and c-Myc to promote gene translation, however, phosphorylation of hnRNP A1 on serine 199 modulated by Akt suppresses the IRES activity (Jo et al., 2008). PRMT5 facilitates the interaction of hnRNP A1 with IRES by methylating R218 and R225 by to promote IRES-dependent translation of cyclin D1 and c-Myc (Gao et al., 2017). These findings suggest that the ITAF activity of hnRNP A1 is influenced not only by its expression level but also by its post-translational modifications (PTMs) status, and the PTMs of hnRNP A1 are crucial regulators of ITAF bioactivities.

In addition to its translation promoting activities, the translational repression effects of hnRNP A1 *via* interaction with IRES have also been reported (Table 3). For example, hnRNP A1 binds to the IRES of cellular apoptotic peptidase activating factor 1 (apaf-1) mRNA to repress its translation (Li et al., 2019). hnRNP A1 interacts with the IRES of X-linked inhibitor of apoptosis (XIAP), suppressing its translation (Lewis et al., 2007). These findings show that hnRNP A1, along with subcellular localization, plays a vital role in regulating IRES-dependent translation.

**TABLE 3:** The transcription and translation of genes repressed by hnRNP A1.

Genes	Binding sites	Functions	Reference
human thymidine kinase (htk)	an ATTT sequence motif in CCRU of the promoter	Repressing Transcription	Lau et al. (2000)
apaf-1	IRES	Repressing Translation	Li et al. (2019)
Nanos	3′ UTR	Repressing Translation	Ji and Tulin, (2016)
XIAP	IRES	Repressing Translation	Lewis et al. (2007)

## hnRNP A1 influences RNA stability

hnRNP A1 influences mRNA stability in addition to alternative splicing, transcription and translation. The mRNA stability and degradation regulatory pathways have been extensively reviewed by Clarke et al. (Clarke et al., 2021). AU-rich elements (ARE) in 3′ untranslated region (UTR) of mRNA function as a potent mRNA destabilizing element (Chen and Shyu, 1995). By binding to the reiterated AUUUA sequence of the 3′-UTR of the lymphokine, *c-myc* and *c-fos* proto-oncogene mRNA in human T lymphocytes, hnRNP A1 enhances the mRNA stability (Hamilton et al., 1993). Furthermore, hnRNP A1 is identified as an ARE-binding protein in 3′UTR of cIAP1, (crucial member of the apoptosis inhibitor family), increasing its mRNA stability under cytotoxic conditions such as UV radiation (Zhao et al., 2009). These findings are suggestive of a cancer-promoting effect of hnRNP A1 through influencing specific mRNA stability. Further research shows that ARE binding ability of hnRNP A1 is negatively correlated with its serine-threonine phosphorylation status (Hamilton et al., 1997). Besides binding to ARE, hnRNP A1 can bind to a putative hairpin-loop region in the 3′ UTR of CYP2A5 mRNA, enhancing the mRNA stability (Glisovic et al., 2003).

miRNAs are endogenous single-stranded RNAs that negatively regulates the targeted mRNAs. The RNase III Drosha enzyme catalyzes the biogenesis of miRNAs, forming the stem-loop precursors, which are then processed by type III ribonuclease Dicer, producing the mature miRNAs. The hnRNP A1 participates in this process and regulates the biogenesis of miRNAs. HnRNP A1 binds to the conserved terminal loop of pri-let-7a-1 and inhibits its processing by Drosha, inhibiting let-7a biogenesis (Michlewski and Caceres, 2010). In contrast, hnRNP A1 promotes miRNA-18a biogenesis mediated by identifying the terminal loop RNA and therefore creating a favorable cleavage site for Drosha, which could be a potential general principle of miRNA biogenesis and regulation (Michlewski et al., 2008; Kooshapur et al., 2018). Furthermore, hnRNP A1 serves as a powerful loading protein for microRNAs in small extracellular vesicle (sEV-miRNAs), facilitating tumor proliferation and migration of non-small cell lung cancer (Li et al., 2021). hnRNP A1 has also shown to mediate the package of miR-196a into cancer-associated fibroblasts (CAF)-derived exosomes targeting CDKN1B and ING5 to endow cisplatin resistance of head and neck cancer (Qin et al., 2019). As a result, based on the contribution of miRNAs to the stability and translation of mRNAs (Fabian et al., 2010), hnRNP A1 can modulate mRNA stability and translation indirectly mediated by miRNAs.

Nonsense-mediated mRNA decay (NMD) is a conserved mRNA quality control mechanism for ensuring the fidelity of gene expression, which is another mRNA degrading pathway targeting the mRNAs that lack of the proper arrangement of translational signals. A large percentage of alternatively spliced transcripts that harbor a premature termination codon (PTC) can be degraded by NMD pathway (Lareau et al., 2007). Alternative polyadenylation (APA) is an alternative splicing process that produces transcript 3′-UTRs with distinct sequences, lengths and stabilities. Alternative polyadenylation of 3′UTR in hnRNP A2/B1 pre-mRNA is induced by increased hnRNP A1, resulting in mRNA degradation *via* the NMD pathway (Patry et al., 2003; McGlincy et al., 2010). However, the role of hnRNP A1 in NMD is still largely unknown, requiring further investigation to identify the underlying mechanisms.

## Available approaches targeting hnRNP A1

The crucial roles of hnRNP A1 have been indicated in cancers (Roy et al., 2017) and neurodegenerative diseases (Clarke et al., 2021). hnRNP A1 selectively regulates mRNA splicing processes, promoting expression of specific protein variants linked to tumorigenesis and cancer progression, and also modulates the transcription and translation of several oncogenes or anticancer genes (Roy et al., 2017). The pathogenesis of neurodegenerative diseases including amyotrophic lateral sclerosis, multiple sclerosis, Alzheimer’s disease, and Huntington’s disease may be influenced by hnRNP A1 dysregulation (Clarke et al., 2021). Therefore, targeting hnRNP A1 for the treatments of the relevant cancers and neurological disorders may be encouraging. Here, we have reviewed the reported approaches targeting hnRNP A1 (Table 4).

**TABLE 4:** Available Approaches Targeting hnRNP A1.

Candidates	Targeting process affected/Checked	Reference
VPC-80051	targeting the RNA-binding domain (RBD) of hnRNP A1	Carabet et al. (2019)
Camptothecin	bind directly to hnRNP A1 and inhibit the hnRNP A1/ topoisomerase I (top I) interaction	Manita et al. (2011)
Riluzole	bind directly to hnRNP A1 and inhibit its ITAF activity	Benavides-Serrato et al. (2020)
Compound 11	block hnRNP A1 from interacting with IRES of c-MYC and cyclin D1	Holmes et al. (2016)
Quercetin	binds to the C-terminal region of hnRNPA1, causing it to be retained in the cytoplasm	(Ko et al., 2014; Tummala et al., 2017)
Idarubicin	impairing the binding between EV71 IRES RNA and hnRNP A1	Hou et al. (2016)
Tetracaine hydrochloride	reduced protein stability of hnRNP A1	Huang et al. (2022)
miR-18a	mRNA degradation	Fujiya et al. (2014)
miR-490	mRNA degradation	Zhou et al. (2016)
miR-206	mRNA degradation	Fu et al. (2020)
miR-424	mRNA degradation	Otsuka et al. (2018)
miR-503	mRNA degradation	Otsuka et al. (2018)
miR-135a-5p	mRNA degradation	Sokol et al. (2018)
miR-149–5p	mRNA degradation	
miR-137	mRNA degradation	Sun et al. (2012)
lncRNA RP11-81H3.2	directly interacts with miR-339, weakening the repression from miRNA to mRNA of hnRNP A1	Chen et al. (2020)
lncRNA ANCR	sponge miR-140–3p to inhibit hnRNP A1 degradation	Wen et al. (2020)
lncRNA XIST	sponge miR-326 to inhibit hnRNP A1 degradation	Ding et al. (2021)
BC15	an hnRNP A1-specific single-stranded DNA aptamer, used as hnRNP A1 inhibitor	Li et al. (2012)
ASO (*SMN*)	target the hnRNP A1 binding ISS of SMN intron 7 enhances SMN2 exon 7 inclusion	Beusch et al. (2017)
SSO (*MTRR*)	block the hnRNP A1 binding ESEs created by c.903 + 469T>C MTRR mutation correcting the splicing and restoring protein activity	Palhais et al. (2015)

With a computer-aided drug discovery approach, Carabet et al. (Carabet et al., 2019) developed an inhibitor named VPC-80051 to target the RNA-binding domain (RBD) of hnRNP A1. The compound could effectively suppress both c-Myc transcription and an alternative splice variant of androgen receptor (AR-V7) generation modulated by hnRNP A1. Camptothecin (CPT) is an anti-tumor natural product, that can bind directly to hnRNP A1 and inhibit the hnRNP A1/ topoisomerase I (top I) interaction (Manita et al., 2011), wherein top I is essential for maintaining DNA helical structure and is important for cancer progression. Benavides-Serrato et al. (Benavides-Serrato et al., 2020) discovered that riluzole could bind directly to hnRNP A1 and inhibit its ITAF activity in glioblastoma *via* a riluzole-bead coupled binding assay. Holmes et al. (Holmes et al., 2016) identified a drug named compound 11 (C11) that blocks hnRNP A1 from interacting with IRES of c-MYC and cyclin D1, in glioblastoma cells. Using affinity chromatography, mass spectrometry and *in vitro* binding experiments, it was discovered that quercetin, a flavonoid, interact directly with hnRNP A1. Quercetin binds to the C-terminal region of hnRNPA1, causing it to be retained in the cytoplasm, and subsequently exerts its anti-cancer effects on prostate cancer cells (Ko et al., 2014; Tummala et al., 2017). Idarubicin, an anthracycline compound used for cancer therapy, has been identified as a broad-spectrum enterovirus replication inhibitor that selectively inhibits impairing the binding between EV71 IRES RNA and hnRNP A1 (168). Our recent work, that tetracaine hydrochloride, a local anesthetic, was found to induce the melanoma cell cycle by downregulating hnRNP A1. We discovered that tetracaine hydrochloride treatment reduced protein stability of hnRNP A1, however the underlying molecular mechanism needs further investigation (Huang et al., 2022). These findings suggest that some small-molecule chemical drugs can inhibit hnRNP A1 activity. However, the application of these drugs targeting hnRNP A1 are facing challenges with targeting specificity, side effects and drug delivery efficiency.

hnRNP A1 activities can be inhibited by various biopharmaceutical approaches. miR-18a is reported to target hnRNP A1 in colon cancer cells, because miRNAs bind to mRNAs and downregulate the transcripts of target genes based on sequence complementarity (Fujiya et al., 2014). The miR-490 binds directly to the 3′-UTR of hnRNPA1 mRNA repressing its translation in gastric cancer cells (Zhou et al., 2016). The tumor suppressor miR-206 directly targets hnRNPA1 to attenuate the Warburg effect and proliferation of colon cancer cells (Fu et al., 2020). In breast cancer, resveratrol induces tumor-suppressive miRNAs miR-424 and miR-503, which suppress breast cancer cell proliferation by downregulating the hnRNP A1 expression (Otsuka et al., 2018). As per the reports, miR-135a-5p, miR-149–5p, miR-137, miR-339 are the other miRNAs that target hnRNP A1 (174–176). Long noncoding RNA (lncRNA) can regulate hnRNP A1 expression level *via* a competing endogenous RNA (CeRNA) mechanism. In gastric cancer, lncRNA RP11-81H3.2 directly interacts with miR-339, weakening the repression from miRNA to mRNA of hnRNP A1 (176). The lncRNA ANCR can sponge miR-140–3p to inhibit hnRNP A1 degradation promoting hepatocellular carcinoma metastasis (Wen et al., 2020). Similarly, lncRNA XIST upregulates hnRNP A1 in Multiple sclerosis (MS) through the XIST-miR-326-HNRNPA1 signaling axis (Ding et al., 2021). However, one miRNA targets more than one mRNA, and one single mRNA can be targeted by multiple miRNAs, translation of miRNAs to clinical practice remains challenging.

Additionally, more approaches targeting hnRNP A1 have been developed. BC15, an hnRNP A1-specific single-stranded DNA aptamer, is used as hnRNP A1 inhibitor, eliciting a strong anticancer effect on the proliferation of cultured hepatoma cells (Li et al., 2012). Anti-sense oligonucleotides (ASO) that target the hnRNP A1 binding ISS of SMN intron 7 enhances SMN2 exon 7 inclusion, reflecting advantages in neuromuscular disease spinal muscular atrophy (Beusch et al., 2017). A similar splice-shifting oligonucleotide (SSO) is utilized to block the hnRNP A1 binding ESEs created by c.903 + 469T>C MTRR mutation correcting the splicing and restoring protein activity (Palhais et al., 2015). Proteolysis targeting chimeras (PROTACs) have recently been proposed for *in vivo* protein degradation by recruiting E3 ubiquitin ligases with high-affinity ligands (Bondeson et al., 2015). In addition, Ghidini et al. (Ghidini et al., 2021) have introduced an updated PROTAC named RNA-PROTACs for effectively degrading RBPs using small RNA mimics docking the RNA-binding site of the RBP, which is a very promising direction for rapidly and selectively targeting RBPs in various diseases.

## Conclusion

**FIGURE 3:**
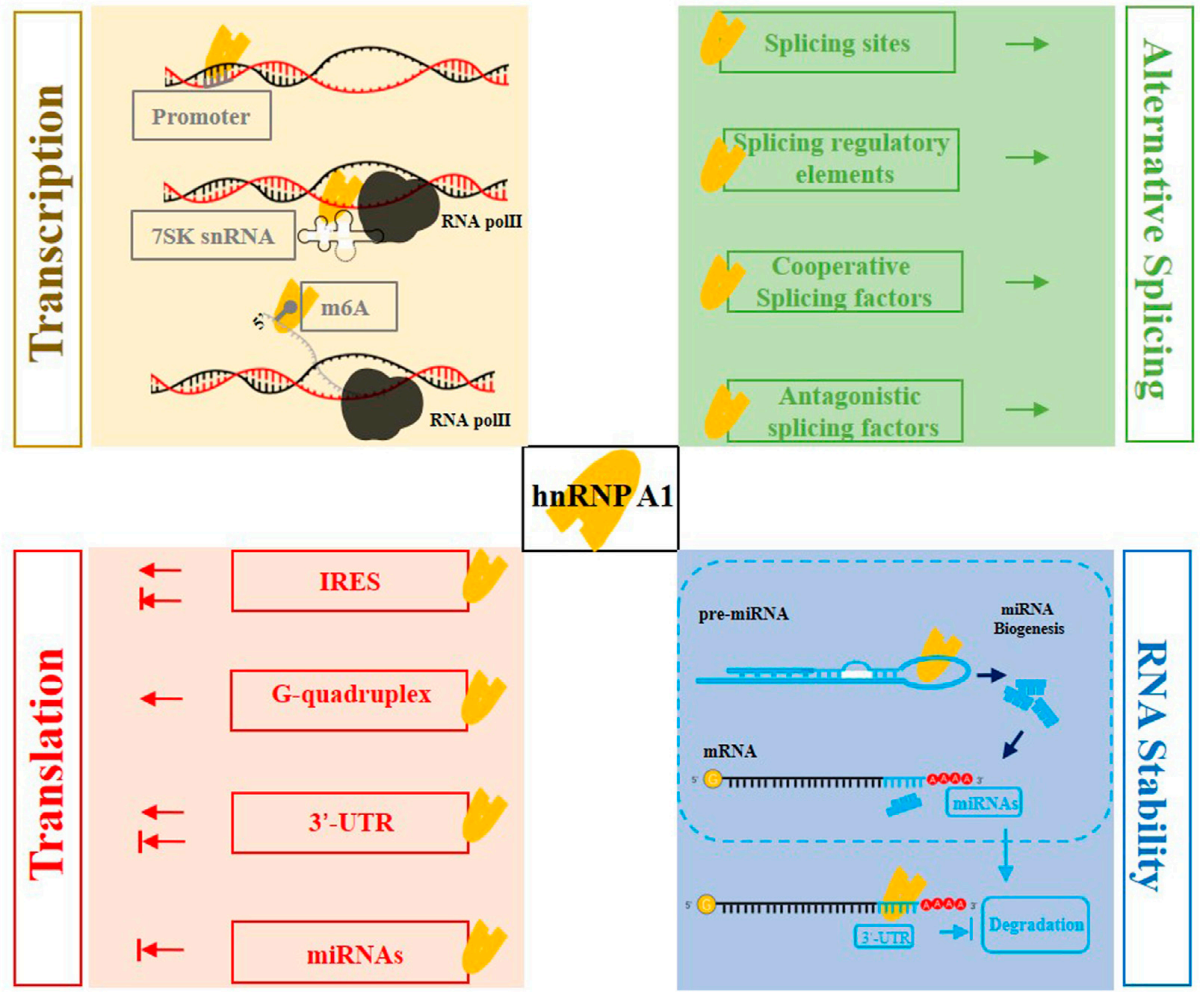
The multifaceted roles of hnRNP A1 in mRNA metabolism. hnRNP A1 affects gene transcription through binding to 7SK snRNA or directly binding to the gene promoter; hnRNP A1 can be recruited to the m6A-modification, and act as a m6A “reader” to promote the transcription. hnRNP A1 enhances mRNA stability through binding to specific sites of 3′-UTR. hnRNP A1 also contributes to the biogenesis of miRNAs, and then the specific miRNAs suppress the mRNA translation or targeting the mRNA decreasing the stability. hnRNP A1 regulates alternative splicing events by binding to specific splice sites and splicing regulatory elements, as well as interacting with cooperative or antagonistic splicing factors. hnRNP A1 increases gene translation by binding to G-quadruplex, and shows positive or negative effects when using its ITAF activity binding to IRES sequence or binding to 3′-UTR of mRNAs.

hnRNP A1 belongs to the hnRNP subfamily and is among the most abundant and widely expressed nuclear proteins. It has multiple functions including participation in transcription regulation, alternative splicing, mRNA translation, miRNA processing and mRNA stability according to its RNA and DNA binding ability (Figure 3). hnRNP A1 can influence the transcription process by directly interacting with the G-quadruplex structure in the promoter, as well as indirectly controlling the transcription of TEFb-dependent genes by influencing transcription elongation by Pol II. hnRNP A1 regulates alternative splicing of multiple genes, collaborating with other cooperative or/and antagonistical splicing factors by binding to splicing sites, splicing regulatory elements of exons or introns, and specific protein domains. HnRNP A1 may exert as ITAF, influencing the IRES-dependent mRNA translation and gene translation by binding to the 3′-UTR of mRNAs. hnRNP A1 can effectively affect the stability of mRNAs *via* binding to specific sites of 3′-UTR, miRNAs biogenesis and NMD pathway. Finally, the targeting hnRNP A1 approaches are reviewed including traditional chemical drugs and biomedicines. According to the evidence, hnRNP A1 plays a crucial role in regulating RNA metabolism, and its dysregulation has been linked to diverse diseases, including cancers and neurodegeneration diseases. As a result, more in-depth exploration in the functions and underlying molecular mechanisms of hnRNP A1 is required, as well as the development of the rapid, selective and highly effective approaches targeting hnRNP A1.
